# Sacubitril/Valsartan: Um Avanço no Tratamento da Insuficiência Cardíaca – Abordando a Qualidade de Vida e a Mortalidade

**DOI:** 10.36660/abc.20230530

**Published:** 2023-09-11

**Authors:** Allan K. N. Alencar

**Affiliations:** 1 Departamento de Engenharia Biomédica Tulane University Nova Orleans EUA Departamento de Engenharia Biomédica – Tulane University, Nova Orleans – EUA

**Keywords:** Insuficiência Cardíaca, Sacubitril/Valsartan/tratamento farmacológico, Qualidade de Vida, Revisão Sistemática, Mortalidade, Metanálise

A insuficiência cardíaca (IC) continua sendo uma das principais causas globais de mortalidade, morbidade e hospitalizações.^1^ Nas últimas duas décadas, o manejo da IC crônica apresentou melhorias significativas com a introdução de novos procedimentos diagnósticos^2^e terapias farmacológicas.^3^ No entanto, alcançar uma melhora clinicamente significativa na capacidade funcional e na qualidade de vida (QV) pode ser mais importante para os pacientes do que simplesmente prolongar a vida.^4^ A IC afeta negativamente a qualidade de vida relacionada à saúde (QVRS) nos domínios físico, mental e social.^5^ Consequentemente, os pacientes com IC apresentam qualidade de vida prejudicada, mesmo quando comparados a indivíduos com outras condições crônicas debilitantes.^6^ Nos tempos contemporâneos, muitos pacientes com IC valorizam tanto a melhoria da QVRS por meio do tratamento quanto o prolongamento de sua expectativa de vida.^7^

Este editorial discute os achados de um trabalho recentemente publicado^8^ que visa revisar a melhora da qualidade de vida com sacubitril/valsartan em pacientes com IC com fração de ejeção reduzida ou preservada (FEVEr/FEVEp) com base em ensaios clínicos prospectivos. Os autores^8^ realizaram uma pesquisa sistemática da literatura no PubMed, Embase e na Cochrane Library para ensaios clínicos randomizados (ECRs) e estudos de coorte prospectivos publicados até julho de 2021. Um total de seis ensaios clínicos e 16.854 pacientes com IC foram incluídos na análise. O desfecho primário foi a alteração da linha de base no Questionário de Cardiomiopatia de Kansas City-Clinical Summary Score (KCCQ-CSS). Os desfechos secundários incluíram pontuações em outros domínios do KCCQ, ocorrência de eventos adversos graves (EAGs) e mortalidade geral.

Os resultados da metanálise demonstraram que o tratamento com sacubitril/valsartan melhorou significativamente o KCCQ-CSS em comparação com o grupo controle. Além disso, sacubitril/valsartan foi associado a uma redução significativa na mortalidade em comparação com o grupo controle. No entanto, nenhuma redução significativa nos EAGs foi encontrada entre os pacientes com IC tratados com sacubitril/valsartan e o grupo controle.

Esta revisão^8^ abrangente fornece informações valiosas sobre os benefícios potenciais do sacubitril/valsartan na melhoria da qualidade de vida de pacientes com IC. A abordagem do estudo, que inclui uma pesquisa sistemática da literatura e metanálise de ensaios clínicos relevantes, confere credibilidade às suas descobertas. Os autores^8^ destacam efetivamente a importância de considerar as melhorias na qualidade de vida como um resultado crucial no tratamento da IC. Como o ônus da IC continua a ser um grande desafio para a saúde, o foco em melhorar o bem-estar e a capacidade funcional dos pacientes é uma direção notável para futuras pesquisas e abordagens terapêuticas.^9^

Um dos pontos fortes do estudo^8^ é a inclusão de múltiplos desfechos, como mortalidade, eventos adversos graves e vários domínios do Questionário de Cardiomiopatia de Kansas City (KCCQ), para avaliar de forma abrangente o impacto do sacubitril/valsartan em pacientes com IC. Os achados demonstram uma redução significativa na mortalidade e uma melhora no KCCQ-CSS, indicando um impacto positivo na qualidade de vida relacionada à saúde dos pacientes. A análise de subgrupos fornece informações adicionais sobre os efeitos diferenciais de sacubitril/valsartan com base na fração de ejeção do ventrículo esquerdo, destacando a necessidade de estratégias de tratamento personalizadas.

No entanto, o estudo^8^ reconhece algumas limitações, como a variação dos instrumentos de avaliação da qualidade de vida na literatura. Embora o KCCQ seja bem validado e amplamente utilizado na pesquisa de IC, a inclusão de outras medidas poderia potencialmente oferecer uma avaliação mais abrangente do bem-estar do paciente.^10^ Além disso, os tamanhos de amostra relativamente pequenos de alguns estudos podem limitar o poder estatístico para resultados específicos.

Apesar dessas limitações, os resultados do estudo são valiosos para médicos e pesquisadores. A melhora significativa

na qualidade de vida relacionada à saúde e redução da mortalidade associada ao tratamento com sacubitril/valsartan fornece evidências convincentes para sua inclusão nas diretrizes de tratamento da IC.^11^ Ao enfatizar os resultados centrados no paciente, esta revisão se alinha com o paradigma em evolução da medicina personalizada, onde as terapias são adaptadas para atender às necessidades e prioridades individuais do paciente.

Em conclusão, este estudo^8^ contribui para o crescente corpo de evidências que suportam o uso de sacubitril/valsartan como uma opção terapêutica valiosa para pacientes com IC. A comunidade de pesquisa deve continuar a explorar seus benefícios, possivelmente em combinação com outras terapias, e identificar os subgrupos de pacientes que mais se beneficiariam com esse tratamento. Como a IC continua a impor uma carga global significativa, adotar abordagens centradas no paciente e considerar as melhorias na qualidade de vida como resultados essenciais sem dúvida contribuirá para melhores resultados e atendimento ao paciente. Mais estudos controlados randomizados bem desenhados com tamanhos de amostra suficientes são necessários para investigar o impacto exato de sacubitril/valsartan na qualidade de vida de pacientes com IC, fornecendo evidências mais robustas para seu uso clínico. Com as descobertas promissoras desta metanálise, os médicos podem considerar a incorporação de sacubitril/valsartan em suas estratégias de tratamento para pacientes com IC para melhorar sua sobrevida, bem-estar geral e capacidade funcional. A ênfase nos resultados centrados no paciente neste estudo marca uma mudança fundamental na abordagem do tratamento da IC, reconhecendo a importância das melhorias na qualidade de vida para melhorar a vida dos pacientes e a satisfação com o tratamento. À medida que os pesquisadores se aprofundam, explorar o impacto econômico do tratamento com sacubitril/valsartan e sua relação custo-benefício em comparação com outras terapias para IC é crucial para informar os tomadores de decisão de saúde e garantir o acesso aos tratamentos mais eficientes para todos os pacientes.

Este estudo^8^ tem o potencial de influenciar a prática clínica e as diretrizes, pois esclarece a importância da melhoria da QVRS como parte integrante do manejo e avaliação do tratamento da IC. À medida que os sistemas de saúde se esforçam para oferecer cuidados baseados em valor, as intervenções que prolongam a vida e melhoram o bem-estar dos pacientes tornam-se fundamentais. Além disso, os achados deste estudo podem estimular discussões entre formuladores de políticas de

saúde e pagadores sobre o reembolso e acessibilidade do sacubitril/valsartan, tornando-o mais acessível a uma população mais ampla de pacientes com IC.

Apesar do progresso alcançado no tratamento da IC, os desafios permanecem na abordagem das disparidades no acesso aos cuidados de saúde e nos resultados para diferentes grupos de pacientes.^12^ Fatores como idade, status socioeconômico, localização geográfica e comorbidades podem afetar significativamente as opções de tratamento e as respostas do paciente.^12^ Pesquisadores e profissionais de saúde devem trabalhar juntos para garantir acesso equitativo a terapias inovadoras como sacubitril/valsartan e considerar as diversas necessidades e preferências dos pacientes com IC. Além disso, promover a educação e o envolvimento do paciente em suas decisões de tratamento pode capacitar os indivíduos a participar ativamente de seus cuidados, levando a uma melhor adesão às terapias prescritas e a melhores resultados a longo prazo.

Como o conhecimento médico continua a evoluir, o monitoramento contínuo e a coleta de dados são essenciais para verificar e refinar os achados deste estudo. A inclusão de resultados relatados pelo paciente e dados qualitativos pode oferecer insights mais profundos sobre as experiências subjetivas e percepções de pacientes com IC recebendo sacubitril/valsartan, complementando os dados quantitativos de ensaios clínicos. Estudos observacionais de longo prazo e evidências do mundo real serão fundamentais para avaliar o impacto sustentado da terapia na QVRS e no bem-estar geral do paciente.

Em conclusão, os achados do artigo^8^ ressaltam a importância do sacubitril/valsartan na melhoria da qualidade de vida e na redução da mortalidade em pacientes com IC. Este estudo^8^ contribui para a crescente evidência que apoia o manejo da IC centrado no paciente e reconhece as melhorias na QVRS como objetivos terapêuticos cruciais. Ao adotar resultados multidimensionais e estratégias de tratamento individualizadas, os profissionais de saúde podem otimizar o atendimento ao paciente e promover maior bem-estar entre os pacientes com IC. Embora este estudo^8^ forneça informações valiosas, mais pesquisas e colaboração são necessárias para entender completamente o potencial do sacubitril/valsartan para enfrentar os desafios multifacetados da IC e melhorar a vida dos pacientes em todo o mundo. Com dedicação contínua à pesquisa, inovação e cuidados centrados no paciente, os profissionais de saúde podem fazer progressos substanciais no combate ao fardo global da IC e melhorar a saúde geral e a felicidade das pessoas afetadas por essa condição.
