# Supplementary material for: Sacubitril/Valsartan: Um Avanço no Tratamento da Insuficiência Cardíaca – Abordando a Qualidade de Vida e a Mortalidade
Source: Arq Bras Cardiol. 2023 Sep 11;120(8):e20230530. [Article in Portuguese] doi: 10.36660/abc.20230530 (PMC12092058; doi:10.36660/abc.20230530)
Supplement: Translation

# Translation: Sacubitril/Valsartan: A Breakthrough in Heart Failure Management – Addressing Quality of Life and Mortality

**Authors:** Allan K. N. Alencar

**Affiliations:** USA Department of Biomedical Engineering, Tulane University, New Orleans, LA – USA

**Keywords:** Heart Failure, Sacubitril/Valsartan/drug therapy, Quality of Life, Systematic Review, Mortality, Meta-Analysis

Heart failure (HF) remains a major global cause of mortality, morbidity, and hospitalizations.^1^Over the past two decades, the management of chronic HF has seen significant improvements with the introduction of novel diagnostic procedures^2^and pharmacological therapies.^3^However, achieving a clinically significant improvement in functional capacity and quality of life (QoL) might be more important to patients than simply prolonging life.^4^HF negatively affects health-related quality of life (HRQoL) in physical, mental, and social domains.^5^Consequently, HF patients experience impaired QoL even when compared to individuals with other debilitating chronic conditions.^6^In contemporary times, many HF patients value the enhancement of HRQoL through treatment as highly as extending their lifespan.^7^

This editorial discusses the findings of a recently published^8^work that aims to review the improvement in quality of life with sacubitril/valsartan in HF patients with reduced or preserved ejection fraction (HFrEF/HFpEF) based on prospective clinical trials. The authors^8^conducted a systematic literature search in PubMed, Embase, and the Cochrane Library for randomized clinical trials (RCTs) and prospective cohort studies published until July 2021. A total of six clinical trials and 16,854 HF patients were included in the analysis. The primary outcome was the change from baseline in the Kansas City Cardiomyopathy Questionnaire-Clinical Summary Score (KCCQ-CSS). Secondary outcomes included scores in other domains of the KCCQ, occurrence of serious adverse events (SAEs), and overall mortality.

The meta-analysis results demonstrated that treatment with sacubitril/valsartan significantly improved the KCCQ-CSS compared to the control group. Moreover, sacubitril/valsartan was associated with a significant reduction in mortality compared to the control group. However, no significant reduction in SAEs was found between HF patients treated with sacubitril/valsartan and the control group.

This comprehensive review^8^provides valuable insights into the potential benefits of sacubitril/valsartan in improving the quality of life of HF patients. The study’s approach, which includes a systematic literature search and meta-analysis of relevant clinical trials, lends credibility to its findings. The authors^8^effectively highlight the significance of considering quality-of-life improvements as a crucial outcome in HF management. As the burden of HF continues to be a major healthcare challenge, focusing on enhancing patients’ well-being and functional capacity is a noteworthy direction for future research and therapeutic approaches.^9^

One of the study’s^8^strengths is its inclusion of multiple outcomes, such as mortality, serious adverse events, and various domains of the Kansas City Cardiomyopathy Questionnaire (KCCQ), to comprehensively assess the impact of sacubitril/valsartan on heart failure patients. The findings demonstrate a significant reduction in mortality and an improvement in the KCCQ-CSS, indicating a positive impact on patients’ health-related quality of life. The analysis of subgroups further provides insights into the differential effects of sacubitril/valsartan based on left ventricular ejection fraction, highlighting the need for personalized treatment strategies.

However, the study^8^does acknowledge certain limitations, such as the variation in assessment tools for quality of life in the literature. While the KCCQ is well-validated and widely used in HF research, the inclusion of other measures could potentially offer a more comprehensive evaluation of a patient’s well-being.^10^Additionally, some studies’ relatively small sample sizes may limit the statistical power for specific outcomes.

Despite these limitations, the study’s findings are valuable for clinicians and researchers. The significant improvement in health-related quality of life and reduced mortality associated with sacubitril/valsartan treatment provides compelling evidence for its inclusion in HF management guidelines.^11^By emphasizing patient-centered outcomes, this review aligns with the evolving paradigm of personalized medicine, where therapies are tailored to meet individual patient’s needs and priorities.

In conclusion, this study^8^adds to the growing body of evidence supporting the use of sacubitril/valsartan as a valuable therapeutic option for HF patients. The research community should continue to explore its benefits, possibly in combination with other therapies, and identify the subgroups of patients that would benefit the most from this treatment. As HF continues to impose a significant global burden, embracing patient-centric approaches and considering quality-of-life improvements as essential outcomes will undoubtedly contribute to better patient care and outcomes. Further well-designed randomized controlled trials with sufficient sample sizes are needed to investigate the exact impact of sacubitril/valsartan on the quality of life of HF patients, providing more robust evidence for its clinical use. With the promising findings from this meta-analysis, clinicians can consider incorporating sacubitril/valsartan into their treatment strategies for HF patients to improve their survival, overall well-being, and functional capacity. The emphasis on patient-centered outcomes in this study marks a pivotal shift in the approach to HF management, recognizing the importance of QoL improvements in enhancing patients’ lives and satisfaction with treatment. As researchers delve deeper, exploring the economic impact of sacubitril/valsartan treatment and its cost-effectiveness compared to other HF therapies is crucial for informing healthcare decision-makers and ensuring access to the most efficient treatments for all patients.

This study^8^has the potential to influence clinical practice and guidelines, as it sheds light on the importance of HRQoL improvements as an integral part of HF management and treatment evaluation. As healthcare systems strive to deliver value-based care, interventions that extend life and enhance patients’ well-being become paramount. Furthermore, the findings from this study could stimulate discussions among health policymakers and payers regarding the reimbursement and accessibility of sacubitril/valsartan, making it more accessible to a broader population of HF patients.

Despite the progress made in HF management, challenges remain in addressing disparities in healthcare access and outcomes for different patient groups.^12^Factors such as age, socioeconomic status, geographical location, and comorbidities can significantly impact treatment options and patient responses.^12^Researchers and healthcare providers must work together to ensure equitable access to innovative therapies like sacubitril/valsartan and to consider the diverse needs and preferences of HF patients. Additionally, fostering patient education and engagement in their treatment decisions can empower individuals to participate in their care actively, leading to better adherence to prescribed therapies and improved long-term outcomes.

As medical knowledge continues to evolve, ongoing monitoring and data collection are essential to verify and refine the findings of this study. Including patient-reported outcomes and qualitative data could offer deeper insights into the subjective experiences and perceptions of HF patients receiving sacubitril/valsartan, complementing the quantitative data from clinical trials. Long-term observational studies and real-world evidence will be instrumental in assessing the therapy’s sustained impact on HRQoL and overall patient well-being.

In summary, the article’s^8^findings underscore the significance of sacubitril/valsartan in enhancing the quality of life and reducing mortality in HF patients. This study^8^contributes to the growing evidence supporting patient-centered HF management and recognizing HRQoL improvements as crucial therapeutic goals. By embracing multidimensional outcomes and individualized treatment strategies, healthcare providers can optimize patient care and foster greater well-being among HF patients. While this study^8^provides valuable insights, further research, and collaboration are needed to fully understand the potential of sacubitril/valsartan in addressing the multifaceted challenges of HF and improving patients’ lives worldwide. With continued dedication to research, innovation, and patient-centered care, healthcare professionals can make substantial progress in combating the global burden of HF and improving the overall health and happiness of those affected by this condition.
